# Letter to the editor regarding “Health economic evaluation of microprocessor and non-microprocessor-controlled prosthetic knees”

**DOI:** 10.33137/cpoj.v8i2.46339

**Published:** 2025-12-31

**Authors:** B Brüggenjürgen, A Riemer, M Gapp

**Affiliations:** Institute for Health Services Research and Technical Orthopedics, Orthopedic Department, Medical School Hannover (MHH) at DIAKOVERE Annastift Hospital, Germany.

**Keywords:** Lower Limb, Amputation, Prostheses, Cost Analysis, Quality of Life, Questionnaire, Mobility, Microprocessor Knee, Cost-effectiveness

Dear Editor,

Real-world evidence plays a crucial role in the assessment of health technologies. In this context, the study by Bosman et al. offers valuable insights by successfully obtaining retrospective Dutch data over a six-month period from lower limb amputees.^1^ Their cross-sectional analysis includes both individuals who underwent acute amputations and those who have been using prostheses for several decades. The granularity of the resource consumption data is notably high, with a meticulously designed questionnaire and expert-derived unit cost estimates. However, it is somewhat unexpected that the incremental cost per Quality-Adjusted Life Year (QALY) reported in their study was higher than that observed e.g. with Nivolumab/Ipilimumab in non–small cell lung cancer, reaching €401,700 per QALY.^2^

In the analysis by Bosman et al., the cost of healthcare resource utilization was found to differ between users of non-microprocessor-controlled (NMPK) and microprocessor-controlled (MPK) knee prostheses, with total costs of €4,981 and €3,909 respectively (excluding the cost of prostheses). The dataset also provided data on the percentage of patients requiring either initial fitting or replacement of their prosthesis during the observed 6-month period, which was reported being at 20% for NMPK users and 16% for MPK users. Hence, the additional average costs associated with these replacements or fittings amount to €883 and €3,363, respectively, for the reported 6-month period.

However, it is important to note that the article does not account for these prorated prosthesis-related costs. Furthermore, the authors suggest that, for each 6-month period, an additional cost factor for a new prosthesis should be considered, which would correspond to a prosthesis replacement cycle of 6 months. This assumption may reflect an error, which, if uncorrected, could significantly overestimate the true costs for individuals requiring and benefitting from high-functionality prosthetic care.

Based on the 6 months figures provided (incremental QALY gain of 0.03, Table 5), we calculate an Incremental Cost-Utility Ratio (ICUR) of €46,916 per QALY gained when comparing MPKs to NMPKs (see Supplementary Material). This is within the thresholds commonly accepted by health technology assessments and is consistent with a broad body of literature in this area.^3,4^

However, even this calculation is based on a relatively short cycle life of the NMPK and MPK prostheses. We approximated the missing information on replacement cycle length by subtracting the proportion of new fittings from the reported number of new prostheses for those having received a replacement only. Based on this approach, we derived replacement cycles of 2.5 years for NMPK and 3.1 years for MPK prostheses (via 1/rate), which may reflect characteristics specific to the patient populations at the two centers. Assuming a still conservative 5-year replacement cycle, the resulting ICUR decreases to a more realistic €19,603 per QALY gained.

We hope this clarification contributes to a more accurate understanding of the economic implications of prosthetic technologies, in particular for decision maker and encourages further sound discussions on the cost-effectiveness of different prosthetic options.

## Supplementary Material


